# Factors affecting tumor ^18^ F-FDG uptake in longitudinal mouse PET studies

**DOI:** 10.1186/2191-219X-3-51

**Published:** 2013-07-10

**Authors:** Wei Sha, Hu Ye, Keisuke S Iwamoto, Koon-Pong Wong, Moses Quinn Wilks, David Stout, William McBride, Sung-Cheng Huang

**Affiliations:** 1 Department of Molecular and Medical Pharmacology, David Geffen School of Medicine at UCLA, Rm. B2-085H CHS, 10833 Le Conte Avenue, Los Angeles, CA 90095, USA; 2 Department of Radiation Oncology, David Geffen School of Medicine at UCLA, Los Angeles, CA 90095, USA; 3 Department of Biomathematics, David Geffen School of Medicine at UCLA, Los Angeles, CA 90095, USA

**Keywords:** F-FDG, PET, Glucose, Tumor, Kinetic

## Abstract

**Background:**

Many biological factors of 2-[^18^ F]fluoro-2-deoxy-d-glucose (^18^ F-FDG) in blood can affect ^18^ F-FDG uptake in tumors. In this study, longitudinal ^18^ F-FDG positron emission tomography (PET) studies were performed on tumor-bearing mice to investigate the effect of blood glucose level and tumor size on ^18^ F-FDG uptake in tumors.

**Methods:**

Six- to eight-week-old severe combined immunodeficiency mice were implanted with glioblastoma U87 (*n* = 8) or adenocarcinoma MDA-MB-231 (MDA) (*n* = 11) in the shoulder. When the tumor diameter was approximately 2.5 mm, a 60-min dynamic ^18^ F-FDG PET scan was performed weekly until the tumor diameter reached 10 mm. Regions of interests were defined in major organs and tumor. A plasma curve was derived based on a modeling method that utilizes the early heart time-activity curve and a late-time blood sample. The ^18^ F-FDG uptake constant *K*_*i*_ was calculated using Patlak analysis on the tumors without an apparent necrotic center shown in the PET images. For each tumor type, the measured *K*_*i*_ was corrected for partial volume (PV), and multivariate regression analysis was performed to examine the effects of blood glucose level ([Glc]) and tumor growth. Corrected Akaike's information criterion was used to determine the best model.

**Results:**

The regression model that best fit the PV-corrected *K*_*i*_ for U87 data was *K*_*i*_/RC = (1/[Glc]) × (0.27 ± 0.027) mL/min/mL (where [Glc] is in mmol/L)**,** and for MDA, it was *K*_*i*_/RC = (0.04 ± 0.005) mL/min/mL, where *K*_*i*_/RC denotes the PV-corrected *K*_*i*_ using an individual recovery coefficient (RC). The results indicated that ^18^ F-FDG *K*_*i*_/RC for U87 was inversely related to [Glc], while [Glc] had no effect on ^18^ F-FDG *K*_*i*_/RC of MDA. After the effects of PV and [Glc] were accounted for, the data did not support any increase of ^18^ F-FDG *K*_*i*_ as the tumor (of either type) grew larger in size.

**Conclusions:**

The effect of [Glc] on the tumor ^18^ F-FDG *K*_*i*_ was tumor-dependent. PV- and [Glc]-corrected ^18^ F-FDG *K*_*i*_ did not show significant increase as the tumor of either type grew in size.

## Background

Small-animal positron emission tomography (PET) imaging is being used increasingly in preclinical research. The use of glucose analogue 2-[^18^ F]fluoro-2-deoxy-d-glucose (^18^ F-FDG) for the *in vivo* measurement of local glucose utilization rate in oncological animal models has been a valuable tool for evaluation of treatment response. Longitudinal imaging allows tracking of the disease progression and provides more sensitive qualitative and quantitative assessments of the effects of an intervention than non-longitudinal measurements [1,2]. Reduction in ^18^ F-FDG uptake from its baseline value is usually used as an indicator of the tumor response to treatment. Standard uptake value (SUV) [3] is commonly used as a measure of glucose utilization activity, but it can be influenced by a variety of biologic and technical factors, including the plasma time-activity curve and blood glucose level [4]. The effect of blood glucose level on ^18^ F-FDG uptake in tumors has been investigated before but with conflicting results. Some found benefit in normalizing SUV by blood glucose level [5-7]; others found no benefit [8-10]. Our previous work also indicated that ^18^ F-FDG uptake in various tissues is affected by blood glucose level differently [11]. It is likely that the value of SUV may not necessarily reflect directly the glucose utilization rate in the tissue of concern, and adjustments for glucose level should not be done indiscriminately.

In this work, we addressed the effect of blood glucose level and tumor growth on both the SUV and ^18^ F-FDG uptake constant (*K*_*i*_) in two different types of tumors. The input function for the calculation of ^18^ F-FDG *K*_*i*_ was derived based on a modeling method that utilizes the early-time dynamic PET images of the heart chamber and a single blood sample taken at the end of the scan [12]; so, the experimental animal did not need to be sacrificed after each scan, and multiple longitudinal studies could be performed. The use of an input function for quantitation of biological function seeks to reduce many effects due to systemic variations.

## Methods

### Tumor models and small-animal imaging

Nineteen 6- to 8-week-old severe combined immunodeficiency (SCID) mice were maintained in a strict defined-flora, pathogen-free environment in the AAALAC-accredited animal facilities at UCLA. Human glioblastoma cell line U87 and breast cancer cell line MDA-MB-231(MDA) were used as a SCID-hu tumor model. Tumor cells were injected (with MDA cells in 11 mice and with U87 cells in 8) subcutaneously as single-cell suspensions in phosphate buffer saline (PBS; about 2 × 10^6^ MDA or about 6 × 10^5^ U87 cells in 100 μL PBS). When the diameter of the tumor grew to approximately 2.5 mm, a PET scan was performed once a week on the same animal until the diameter of the tumor exceeded 10 mm. Tumor size was measured weekly with a caliper, and the volume was calculated as 0.5 × length × width^2^.

Small-animal PET scans were performed either on a microPET Focus 220 scanner running microPET Manager 2.4.1.1 or on an Inveon dedicated PET running IAW 1.5 (Siemens Preclinical Solutions, Knoxville, TN, USA), but the same scanner was used for multiple longitudinal PET scans of each mouse. These scanners provide the same SUV and % ID values for mice imaged sequentially in both systems (unpublished data). List-mode PET data were acquired for 60 min immediately after ^18^ F-FDG injection via a tail vein catheter (18.28 ± 1.19 MBq, approximately 60 μL) in a bolus. Frame durations of all the PET studies were 4 × 1 s, 15 × 0.5 s, 1 × 2 s, 1 × 4 s, 1 × 6 s, 1 × 15 s, 3 × 30 s, 1 × 60 s, 1 × 120 s, 3 × 180 s, 3 × 900 s, and 1 × 51 s. After the PET scan was completed, a 10-min CT scan was acquired with a small-animal CT scanner (MicroCAT II, Siemens Preclinical Solutions, Knoxville, TN, USA) for attenuation correction of the PET measurements.

Tail vein blood glucose levels (in mmol/L) were measured using a blood glucose meter (Abbott AlphaTRAK, Abbott Laboratories, Abbott Park, IL, USA) at the beginning and the end of each scan. A single blood sample (approximately 10 to 15 μL) was collected with a 1-cc syringe from the heart at the end of the study (approximately 70 to 80 min). The whole blood in the syringe was released to a pre-weighed test tube and weighed, and the radioactivity was counted in a gamma counter (WIZARD 3"; PerkinElmer Life Sciences, Turku, Finland).

All animal experiments were performed in accordance with institutional guidelines and protocols approved by the Animal Research Committee of the University of California, Los Angeles, USA.

### Image analysis

Image analysis was performed using AMIDE (http://amide.sourceforge.net/). The 3D isocontour regions of interest (ROIs) were manually defined for the skeletal muscle of the left ventricle (LV), forelegs, liver, and tumor in each mouse on the last 15-min images (about 60 min post ^18^ F-FDG injection). Only tumors without an apparent necrotic center on the ^18^ F-FDG PET images were included in the analysis. The time-activity curves (TAC) in each tissue of interest were calculated. Experimental information for all studies is available online at the UCLA Mouse Quantitation Project website (http://dragon.nuc.ucla.edu/mqp/index.html).

For semi-quantitative analysis, SUV was calculated using the mean voxel value within the ROI of the last 15-min frame (Equation 1). SUVs of each study were recorded and were used for generating the TACs.

(1) $$\mathrm{SUV}=\frac{\mathrm{Radioactivity}\;\mathrm{concentration}\;\mathrm{in}\;\mathrm{ROI}\;\left(\mathrm{MBq}/\mathrm{mL}\right)}{\mathrm{Injected}\;\mathrm{dose}\;\left(\mathrm{MBq}\right)/\mathrm{Weight}\;\mathrm{of}\;\mathrm{the}\;\mathrm{animal}\;\left(g\right)}$$

### Plasma input function

The plasma TAC (TAC_p_; the input function) was derived based on the method reported by Ferl et al. [12]. The method included the use of the early-time LV TAC (*t* < 1 min) (with corrections of delay, dispersion, partial volume effects, and red blood cell uptake) and one whole blood sample taken at the end of the study (approximately 70 min). For each study, a time-dependent plasma-to-whole blood ^18^ F-FDG equilibrium ratio, *R*_PB_(*t*) (Equation 2), was used to convert the whole blood ^18^ F-FDG concentrations to those in plasma [11].

(2) $$R_{P}{}_{B}\left(t\right)=0.386e^{-0.191t}+1.165,$$

where *t* is time in minutes after tracer injection. The input function was assumed to be describable with four exponential components (Equation 3):

(3) $$\begin{array}{c} {\mathrm{TAC}}_{p}=A_{1}e^{-\mu_{{}_{1}}t}+A_{2}e^{-\mu_{{}_{2}}t}+A_{3}e^{-\mu_{{}_{3}}t}\\ -\left(A_{1}+A_{2}+A_{3}\right)e^{-\mu_{{}_{4}}t}. \end{array}$$

The sum of the first three exponential terms was used to describe the main part of the input function; the fourth exponential term was needed so that TAC_p_ was equal to 0 at time 0. All parameters were estimated by simultaneously fitting the plasma ^18^ F-FDG blood curve with Equation 3 and the muscle and liver TACs with two separate 4 K compartmental ^18^ F-FDG models as described by Ferl et al. [12]. The kinetic modeling program SAAM II [13] was used to solve the systems of differential equations and estimate parameters. The Bayesian maximum *a posteriori* parameter estimation in SAAM II was used to improve parameter identification and the accuracy of the predicted input function as described by Ferl et al. [12].

### Kinetic analysis

Both the PET image and blood data were converted to absolute radioactivity concentration (Bq/mL) using a cross-calibration factor derived from cylinder phantom experiments. The ^18^ F-FDG uptake rate constant *K*_*i*_ (*K*_*i*_ = *K*_1_*k*_3_/(*k*_2_ + *k*_3_)) was estimated via the Patlak graphical analysis [14] using the derived plasma input function and tumor TAC data by taking the slope of the linear portion from 15 to 60 min of the plot based on Equation 4:

(4) $$\frac{C_{T}\left(t\right)}{C_{P}\left(t\right)}=\left(\frac{K_{1}k_{3}}{k_{2}+k_{3}}\right)\frac{\int_{0}^{t}C_{P}\left(\tau \right)}{C_{P}\left(t\right)}+\mathrm{Int}$$

(5) $$\mathrm{Int}=\left(\frac{k_{2}}{k_{2}+k_{3}}\;\right)\frac{C_{1}\left(t\right)}{C_{P}\left(t\right)}+\frac{V_{B}C_{B}\left(t\right)}{C_{P}\left(t\right)},$$

where *C*_T_(*t*) is the total ^18^ F-FDG concentration in the tissue of interest, *C*_1_(*t*) is the free ^18^ F-FDG concentration in the tissue, *C*_p_(*t*) is the ^18^ F-FDG concentration in the plasma, *C*_B_(*t*) is the blood ^18^ F-FDG concentration in the vasculature, and *V*_B_ is the volume fraction of blood in the tissue. The early-time tissue data (for *t* < 15 min) were not used because only after an equilibration time (t^*^) will the Patlak plot become linear. The metabolic rate of glucose (MRGlu) was calculated as MRGlu = *K*_*i*_ × [Glc]/LC [15], where [Glc] was the averaged blood glucose level of the two measurements at the beginning and the end of the scan, and LC is the lumped constant. Values of the LC were assumed to be 1.4 for U87 [16] and 1.0 for MDA. The intercept value (Int) shown in Equation 5 is related to the *V*_B_ and the distribution volume of the tracer in the reversible tissue compartment.

### Data analysis and functional relationship determination

*K*_*i*_ and SUVs were partial volume (PV)-corrected by dividing the values by the recovery coefficient (RC) of the tumor. As a first-order approximation, RC for a tumor was calculated based on a sphere of diameter = $\sqrt[{3}]{\frac{6\;\times \;\text{Tumor volume}}{\pi }}\;,$the spatial resolution of the microPET scanner, and an assumed background activity level of 10% of the activity level in the sphere. Four models (Equations 6 to 9) were tested to fit the relationship of glucose concentration and tumor diameter to quantitative PET measures, *Y* (SUV/RC or *K*_*i*_/RC).

(6) Y=a.

(7) $$Y=a+c\times \left[\mathrm{tumor}\;\mathrm{diameter}\right].$$

(8) $$Y=\frac{a}{b+\left[\mathrm{Glc}\right]}$$

(9) $$Y=\frac{\left(a+c\times \left[\mathrm{tumor}\;\mathrm{diameter}\right]\right)}{b+\left[\mathrm{Glc}\right]}$$

The parameters *a* and *c* were to account for the effect due to tumor growth in size; *b* was equivalent to the half saturation glucose concentration of ^18^ F-FDG uptake [11]. Models were tested separately for U87 and MDA. To account for possible effects introduced through repeated measures, models were fit using standard least-squares method as well as mixed-effects method [17]. For models 3 and 4 (Equations 8 and 9), if fitted values for parameter *b* were not found to be significant, the model was rebuilt, setting *b* equal to 0. Mixed-effects models were built assuming no within-group covariance of random effects. For models with more than one fitted parameter, all possible combinations of random effects were tested. If more than one mixed-effect model showed significant improvement over the standard fixed-effect model, the optimal mixed-effect model was chosen by the likelihood ratio test if models were nested or by the corrected Akaike's information criterion (AIC_c_), otherwise. After a fitting method for each model was chosen, the optimal model for each cell line and response variable was chosen based on AIC_c_. Fittings and model comparison were done using the nlme package of the statistical software R [18].

## Results

**Figure 1:**
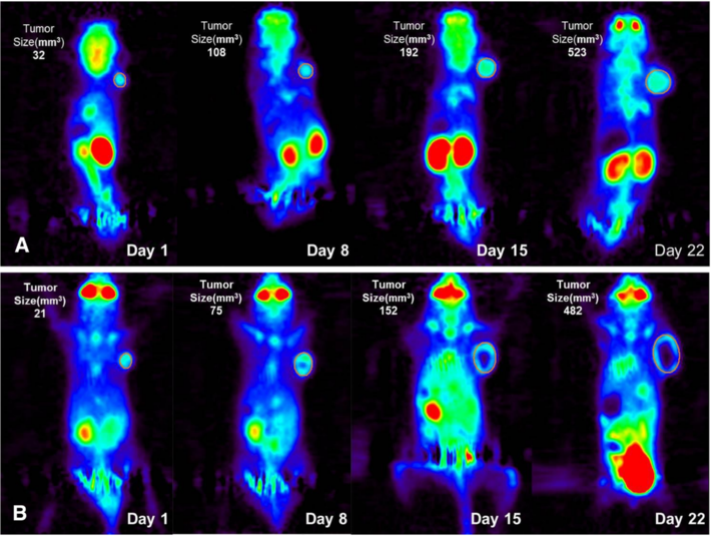
**Longitudinal**^**18**^ **F-FDG PET images of a mouse.** With **(A)** U87 and **(B)** MDA-MB-231 implanted in the shoulder region. In the graphs, day 1 is the date when the first PET scan was performed.

Examples of the coronal tomographic images of weekly microPET scans of a mouse implanted with MDA or U87 tumor cells are shown in Figure 1. The tumors were barely detectable on microPET images by days 28 and 37 after implantation for U87 and MDA, respectively. For MDA tumors, with 33 imaging studies in total, a necrotic core was observed in 17 cases as early as the tumor diameter reached 5 mm, while for U87, with 27 imaging studies in total, a necrotic core was observed only in 5 cases with tumor diameters >8 mm. At the beginning time of each PET scan, the averaged blood glucose levels were 6.88 ± 2.52 and 7.79 ± 2.52 mmol/L, respectively, for MDA- and U87-bearing mice. A modest increase of the [Glc] value was observed at the end of the scan (MDA 8.19 ± 2.25 mmol/L, U87 8.45 ± 2.37 mmol/L), probably due to the effect of isoflurane anesthesia [19].

### Tumor size

**Figure 2:**
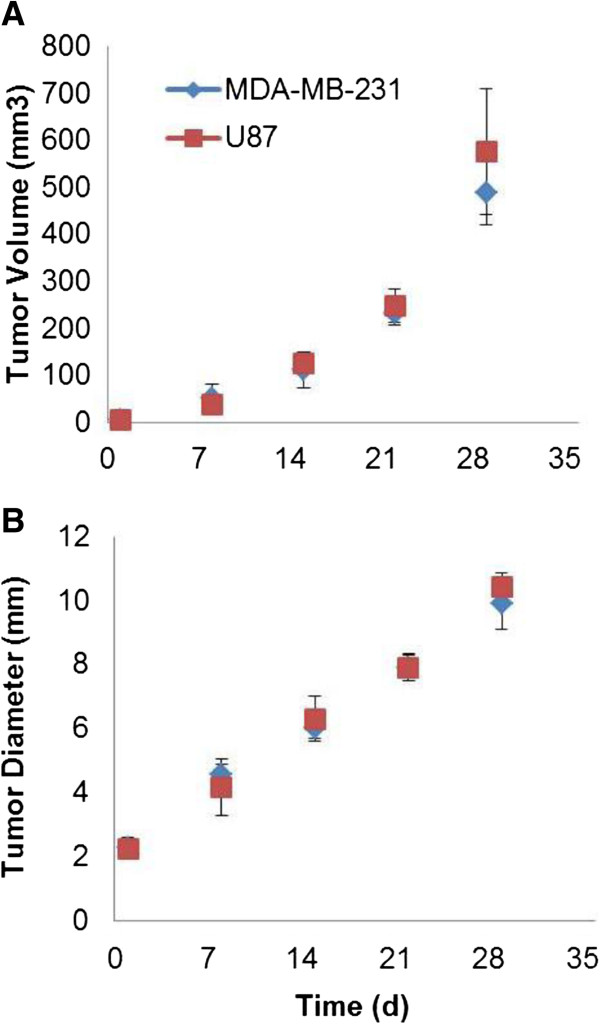
**Tumor volume measurements and calculated diameter throughout the experiment.** The **(A)** tumor volume measurements and **(B)** calculated diameter are for MDA-MB-231 and U87 mice. Day 1 is the date when the first PET scan was performed. Error bars represent 1 SD.

The tumor volume and calculated diameter, respectively, at the time of the first PET scan were 6.06 ± 2.45 mm^3^ and 2.26 ± 0.33 mm for the MDA group and 5.88 ± 2.64 mm^3^ and 2.23 ± 0.34 mm for the U87 group. Tumor volumes continued to increase over time as shown in Figure 2. Tumor diameter appeared to grow linearly with time (Figure 2B). There was no significant difference (*P* > 0.05) in tumor growth rate between the two tumor types (Figure 2). The largest difference between the tumor sizes of the two tumors was at about 30 days after the first PET scan, but the difference was not significant (*P* > 0.05).

### Tumor ^18^ F-FDG uptake and metabolic parameters

Partial volume effect (PVE) of ^18^ F-FDG uptake in tumors with a necrotic core was more difficult to account for since the tumor ^18^ F-FDG uptake became heterogeneous after the necrotic core development. For evaluation of the effects of blood glucose level and tumor growth, only scans without tumor necrotic cores were investigated in this work. SUV was used for semi-quantitative analysis and model fitting to estimate the model parameter values. SUV was found to significantly correlate with the Patlak analysis-derived *K*_*i*_ (*P* < 0.001 for both tumors) and with MRGlu (*P* < 0.001 for both tumors).

**Figure 3:**
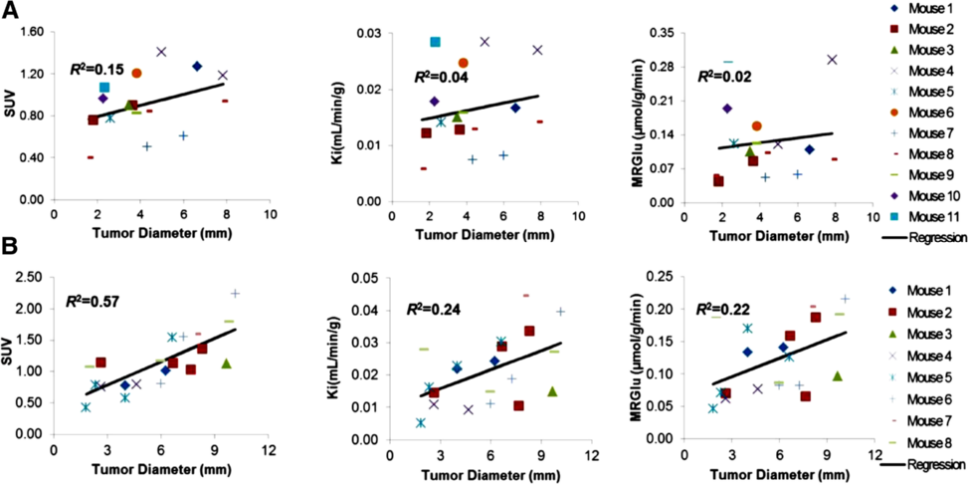
**Relationships versus tumor diameter for SUV, *K***_***i***_**, and MRGlu. (A)** MDA-MB-231 (top row). **(B)** U87 (bottom row).

**Figure 4:**
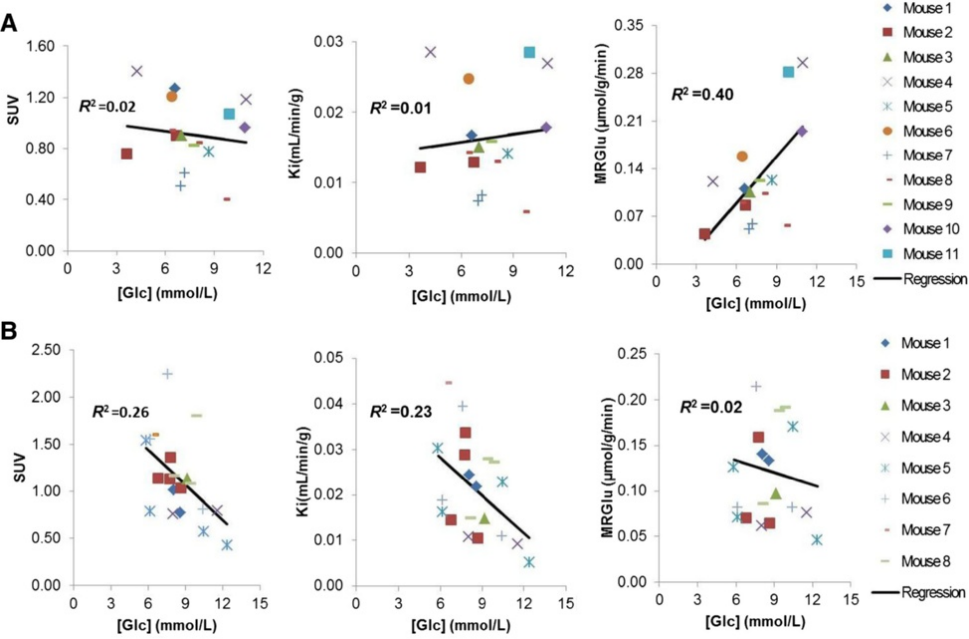
**Relationships versus blood glucose level for SUV, *K***_***i***_**, and MRGlu. (A)** MDA-MB-231 (top row). **(B)** U87 (bottom row).

The relationships as a function of tumor diameter for tumor SUV, *K*_*i*_, and MRGlu are shown in Figure 3 for MDA and U87 tumors. Linear regression analysis was made based on the data pooled from studies with non-necrotic tumors. There was a significant positive correlation between SUV, *K*_*i*_, or MRGlu, and tumor size for U87 (SUV *R*^2^ = 0.57, *P* < 0.001; *K*_*i*_*R*^2^ = 0.24, *P* = 0.027; MRGlu *R*^2^ = 0.22, *P* = 0.039), while no significant corresponding correlations were found for MDA (SUV *R*^2^ = 0.15, *P* = 0.13; *K*_*i*_*R*^2^ = 0.035, *P* = 0.49; MRGlu *R*^2^ = 0.017, *P* = 0.63). For the blood glucose effect on SUV, *K*_*i*_, and MRGlu (Figure 4), neither tumor SUV nor *K*_*i*_ was found to be correlated with the blood glucose level for MDA (with both slope of regression lines and *R*^2^ not significantly different from 0, *P* > 0.05). However, for U87, there was a modest dependency (*P* < 0.05 for both SUV and *K*_*i*_) on blood glucose levels. MDA MRGlu was positively correlated with blood glucose levels (*R*^2^ = 0.39, *P* = 0.009), while no significant correlation was found between MRGlu and blood glucose levels for U87 (*P* > 0.05).

The Patlak analysis also showed that U87 tumors had on average a larger Int (Equation 5) value (0.50 ± 0.13 for U87 versus 0.33 ± 0.05 for MDA, *P* < 0.001). Since Int reflects the fractional blood volume *V*_B_ and the distribution volume of the tracer in the reversible tissue compartment (Equation 5), the higher Int value of U87 signified higher vascular volume and larger *K*_1_ of ^18^ F-FDG in U87, a possible indication of more angiogenesis, than in MDA tumors.

### PV-corrected ^18^ F-FDG uptake versus blood glucose level and tumor size

The RC calculated for PV correction ranged from 0.34 to 0.93 when the sphere radius was from 0.5 to 10 mm. PV-corrected SUV and ^18^ F-FDG *K*_*i*_ in the tumor were fitted by the functions of Equations 6 to 9. Results of model fittings and model comparison are shown in Tables 1 and 2. All of the models are derived from the mouse study showing a tumor with a diameter of >2.5 mm. In the case of U87, for all models and both response variables (i.e., SUV and *K*_*i*_), mixed-effects modeling did not have a significant improvement over standard fitting (Table 1), where residual sum of squares (RSS) and AIC_c_ are calculated based on the non-mixed-effects models. Likelihood ratio tests in Table 1 represent the mixed-effect model with the smallest *P* value. Additionally, for both response variables, models 3 and 4 (Equations 8 and 9) did not return significant values for the parameter *b* in either fixed- or mixed-effects models. Therefore, data in Table 1 represent models built with *b* set to 0. For both response variables, model 3 was ranked as optimal based on AIC_c_.

**Table 1:** Likelihood ratio test on analysis methods and model selection for U87

**Response variable**	**Model**^**a**^	**Log likelihood**		**Random parameters**	***D***^**d**^	***P***^**d**^	**AIC**_**c**_	**RSS**
		**Standard**^**c**^	**Mixed**^**c**^					
*K*_*i*_/RC	1	54.81	54.81	*a*	1.4 × 10^−8^	>0.99	−105.41	4.88 × 10^−3^
	2	55.00	54.82	*a*, *c*	0.368	0.947	−102.92	4.87 × 10^−3^
	3^b^	55.91	55.91	*a*	1.03 × 10^−8^	>0.99	−107.12	4.36 × 10^−3^
	4^b^	56.14	56.14	*a*	1.03 × 10^−8^	>0.99	−102.78	4.27 × 10^−3^
SUV/RC	1	−15.45	−15.45	*a*	4.3 × 10^−9^	>0.99	35.12	5.49
	2	−15.29	−14.53	*a*, *c*	1.53	0.675	37.29	5.41
	3^b^	−13.10	−13.04	*a*	0.102	0.75	30.41	4.34
	4^b^	−13.07	−13.04	*a*	0.0606	0.81	32.85	4.33

^a^Models 1 to 4 correspond to Equations 6 to 9. ^b^These models were built with *b* set to 0. ^c^Standard denotes the standard least-squares method, and mixed denotes the mixed-effects method. ^d^*D* is the likelihood ratio statistic, and *P* is the significance level.

**Table 2:** Likelihood ratio test on analysis methods and model selection for MDA

**Response variable**	**Model**^**a**^	**Log likelihood**		**Random parameters**	***D***^**c**^	***P***^**c**^	**AIC**_**c**_	**RSS**
		**Standard**^**b**^	**Mixed**^**b**^					
*K*_*i*_/RC	1	45.13	48.59	*a*	7.16	0.0075	−90.9	1.46 × 10^−4^
	2	45.79	49.32	*a*	7.06	0.0079	−88.64	1.31 × 10^−4^
	3	31.69	31.69	*a*, *b*	2.58 × 10^−5^	>0.99	−56.46	0.0178
	4	32.83	32.83	*a*, *b*	2.29 × 10^−9^	>0.99	−55.67	0.015
SUV/RC	1	−11.75	−9.779	*A*	3.95	0.047	26.48	0.591
	2	−10.562	−6.5524	*a*, *c*	8.02	0.0456	28.73	0.129
	3	−31.25	−31.25	*a*	2.54 × 10^−5^	>0.99	69.42	46.57
	4	−28.28	−28.28	*a*	7.07 × 10^−6^	>0.99	66.56	32.14

^a^Models 1 to 4 correspond to Equations 6 to 9. ^b^Standard denotes the standard least-squares method, and mixed denotes the mixed-effects method. ^c^*D* is the likelihood ratio statistic, and *P* is the significance level.

In the case of MDA (Table 2), for both response variables, mixed-effects modeling showed a significant improvement over standard fitting in models 1 (Equation 6) and 2 (Equation 7). RSS and AIC_c_ were thus calculated based on the mixed-effects models. For models 3 and 4, they were based on the non-mixed-effects models because incorporation of mixed effects did not lead to a significant improvement over standard fitting. For both response variables, model 1 was ranked as optimal based on AIC_c_.

**Figure 5:**
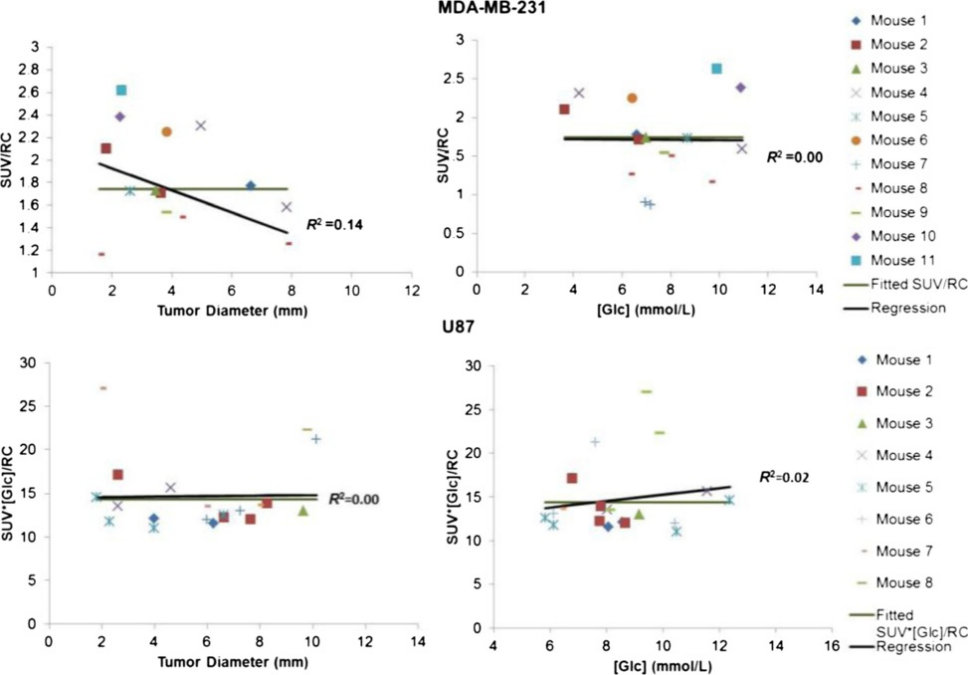
**Relationships of corrected SUV versus tumor size and blood glucose level.** Top row, MDA-MB-231; bottom row, U87.

**Figure 6:**
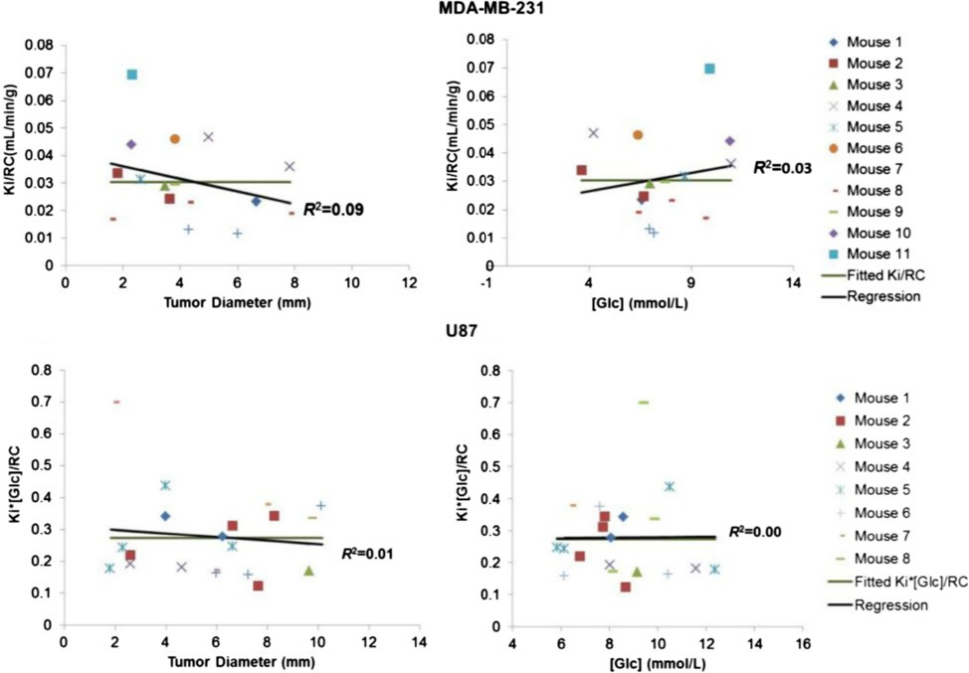
**Relationships of corrected *K***_***i ***_**versus tumor size and blood glucose level.** Top row, MDA-MB-231; bottom row, U87.

For the MDA cell lines, the best models were SUV/RC = 1.8 ± 0.15 (*P* < 10^−5^), with random effect SD = 0.414, and *K*_*i*_/RC = 0.04 ± 0.005 mL/min/mL (*P* < 10^−5^), with random effect SD = 0.014. For the U87 cell lines, the best models were SUV/RC = (1/[Glc]) × (14.36 ± 0.85), (*P* < 10^−5^), and *K*_*i*_/RC = (1/[Glc]) × (0.27 ± 0.027) mL/min/mL, (*P* < 10^−5^). After both partial volume and blood glucose concentration were accounted for (MDA: corrected SUV = SUV/RC and corrected *K*_*i*_ = *K*_*i*_/RC; U87: corrected SUV = (SUV × [Glc])/RC and corrected *K*_*i*_ = (*K*_*i*_ × [Glc])/RC), the data did not support any significant change of SUV or *K*_*i*_ when the tumor (of either cell line) grew bigger in size (but before necrotic core development) as shown in Figures 5 and 6 (corrected SUV: *R*^2^ = 0.14 and 0.0009 for MDA and U87, respectively; corrected *K*_*i*_: *R*^2^ = 0.09 and 0.02 for MDA and U87, respectively, *P* > 0.05 for all situations). The corrected SUV or corrected *K*_*i*_ was also found to be uncorrelated to blood glucose concentrations with the slope of regression lines not significantly different from 0 (corrected SUV: *R*^2^ = 0.0006 and 0.04 for MDA and U87, respectively; corrected *K*_*i*_: *R*^2^ = 0.03 and 0.003 for MDA and U87, respectively, *P* > 0.05 for all situations).

## Discussion

In this work, a set of longitudinal quantitative ^18^ F-FDG PET studies was performed on tumor-bearing mice, and a method to account simultaneously for the blood glucose level and tumor growth has been tested. While the impact of medium/blood glucose levels on ^18^ F-FDG uptake in tumors has previously been investigated in both *in vitro* and *in vivo* studies [20,21], no longitudinal studies have been reported to elucidate the associations of both blood glucose level and tumor size with ^18^ F-FDG *K*_*i*_ in tumors. Our quantitative analysis based on ^18^ F-FDG uptake, kinetics analysis, and model selection addresses the effect of blood glucose level and tumor size (for tumors without necrotic cores) in two different types of tumors. The results suggested that the need to account for the confounding effects of glucose in the fitting of measured data is tumor cell line dependent and that appropriately taking this into account would lead to improvement in the measurement sensitivity of the true biological signals in the tumors.

Results of the growth rate and Patlak analysis showed that human breast cancer MDA cells have an adequate proliferation rate in SCID mice for longitudinal ^18^ F-FDG PET studies, as do human glioma U87 cells. In this work, tumor growth was monitored longitudinally, and it was found that MDA and U87 had a similar growth rate for about 30 days after the baseline imaging time point (Figure 2). However, MDA developed necrotic cores much earlier than U87 (Figure 1), probably due to different angiogenesis rates between the two tumor types (U87 > MDA). The larger Int value derived from the U87 tumor ^18^ F-FDG kinetics is consistent with having more angiogenesis in U87. Although a larger proportion of tumor cells in tissue would increase the FDG uptake rate constant and thus the slope of the Patlak plot, it is not expected to affect the value of Int by a large amount. The larger value of Int also supports the reasoning that the higher angiogenesis rate is the cause of the observed slower development of necrotic core in the U87 tumors as compared to the MDA tumors.

In PET tumor imaging, SUV is the most frequently used index for characterizing tumor ^18^ F-FDG uptake. However, the value of its use for interpreting ^18^ F-FDG PET scans was found to be limited because of the considerable overlap between SUV measurements in malignant and benign lesions and subtle changes at early response to therapy [22]. Therefore, large variability in SUV measurements could affect the clinical interpretation. Generally speaking, these variability sources can be categorized into two types. One comes from biological factors (e.g., tumor's size/configuration changes, plasma substrate level, insulin level, and input function variations). Another type is associated with technical factors (e.g., reconstruction parameter changes). Our study was designed to examine the effect of blood glucose level and tumor growth on ^18^ F-FDG uptake.

Results of MDA showed that SUV, *K*_*i*_, and MRGlu were relatively constant when the tumor grew bigger (Figure 3); no correlation was found between SUV or *K*_*i*_, and blood glucose levels, but MRGlu increased with increasing blood glucose levels (Figure 4). Unlike that in MDA, SUV and *K*_*i*_ in the U87 tumor correlate significantly with tumor size and blood glucose levels, indicating that correction for both blood glucose level and tumor size is needed in the calculation of SUV and *K*_*i*_ to reflect tumor glucose utilization rate. These results also suggested that the glucose uptake regulation could be different in different tumor types, and correcting for blood glucose level or not in the SUV formula should be done selectively. Reported results in the literature on the effect of blood glucose levels on a few other types of tumors were somewhat controversial. Most studies have demonstrated that ^18^ F-FDG uptake into human cancer cells is inhibited by increasing blood glucose levels because of saturation of glucose uptake in tumors [6,7]. Our results from U87 data were consistent with these findings. However, animal studies performed by Torizuka et al. [10] indicated that the uptake of ^18^ F-FDG in mammary carcinoma was reduced for insulin-induced hypoglycemia. In our present study, ^18^ F-FDG SUV in the mammary cancer MDA has a slightly negative slope but with an insignificant correlation, with respect to blood glucose levels (Figure 4).

When tracer uptake in small tumors was measured, large biases could be introduced by the PVE [23]. We used the calculated RC to reduce the errors attributable to PVE. Four models (Equations 6 to 9) with one to three independent parameters were used to account for glucose level and to evaluate if the PV-corrected ^18^ F-FDG SUV and *K*_*i*_ changed as the tumors grew in size. Likelihood ratio tests (Tables 1 and 2) showed that mixed-effects modeling is not necessary for the U87 data but is necessary for the MDA cell line, suggesting that the U87 has more homogenous development than the MDA among different mice. This is consistent with the larger data scatter of ^18^ F-FDG uptake obtained for the MDA cell line than for U87 in *in vitro* experiments using multiwell plates [24]. The best model (Equation 8) for U87 indicates that U87 *K*_*i*_ was inversely correlated to the blood glucose levels. That is similar to our previous findings on ^18^ F-FDG *K*_*i*_ in the brain [11] that showed a significant inverse relationship between cerebral ^18^ F-FDG *K*_*i*_ and the blood glucose level. The best model (Equation 1) for MDA is consistent with the results of our *in vitro* studies, which showed that the ^18^ F-FDG uptake in MDA was affected little by medium glucose level over the normal physiological glucose range [24]. Since U87 is a human cancer cell line originating from the brain, the finding that U87 behaves like the brain tissue in glucose metabolism is interesting, but not unexpected. The ^18^ F-FDG SUV and *K*_*i*_, after accounting for the PVE and blood glucose levels (Figures 5 and 6), did not show any significant correlation with tumor size, further confirming that tumor glucose utilization rate was rather stable when the tumor (of either MDA or U87) grew bigger in size (but before necrotic core development). The relationship indicates that without treatment intervention, tumor cells/tissues do not change their metabolic aggressiveness as tumors grow (at least before necrosis appears).

The current results showed that the use of either SUV or *K*_*i*_ gave the same conclusion regarding the effects of blood glucose level and tumor growth on glucose utilization rates for MDA and U87. The use of *K*_*i*_, however, was supposed to better account for the systemic variations in ^18^ F-FDG delivery between studies or animals. In the present study, these variations were small because the animals were obtained from the same source, had the same diet and similar age, and were treated similarly. When the variations in the systemic condition are large, as in a patient population, the use of SUV as an indicator of tumor glucose utilization rate is expected to give a larger variability than that of *K*_*i*_ and may not be as sensitive to reveal the same effects. Similar advantages of *K*_*i*_ over SUV were also observed in one of our previous studies on the effects of blood glucose level on the brain ^18^ F-FDG uptake [11]. The main desirable feature of SUV is that it does not need dynamic PET imaging and is thus more practical for human studies.

The present results indicate that ^18^ F-FDG *K*_*i*_ in MDA and U87 differs considerably in response to altered blood glucose level, suggesting that regulation of ^18^ F-FDG uptake and glucose metabolism are tumor-type dependent. However, more work needs to be done to account for effects due to other possible factors, including blood insulin level and tumor heterogeneity (when a necrotic core appears), to further improve the reliability of the quantitative measurements and to examine the metabolic stability/change after a necrotic core develops. Insulin is known to induce a host of effects on traditional metabolic actions such as glucose transport and utilization [25]. Insulin also has vascular-specific actions that are particular to vascular tissues [26]. Although the effects of insulin on tumor ^18^ F-FDG uptake have been studied by others before [10,27], the prior studies all involved insulin injections into the animal or patient that yielded a non-steady state circulating insulin level and also a variable blood glucose level at the same time. As a result, ^18^ F-FDG uptake could be affected by either or both factors and is more difficult to interpret. Only after the known confounding factors are properly accounted for can one begin to critically assess the temporal changes in ^18^ F-FDG uptake kinetics during normal growth or due to intervention or treatment.

A frequently asked question about ^18^ F-FDG uptake in tumors is whether it reflects more tumor biology versus inflammatory response associated with the xenograft. Our present study would not be able to provide a definitive answer. Although inflammatory response is usually considered to be transient, it could be compensated by tumor biology changes. A longitudinal PET study with coordinated tissue autopsy examination could be performed in the future to address this question.

## Conclusions

Results from the present study showed that ^18^ F-FDG *K*_*i*_ in the U87 tumor varies inversely with the blood glucose levels. In contrast, MDA ^18^ F-FDG *K*_*i*_ is independent of the blood glucose level, indicating that different transporter/hexokinase adaptations to blood glucose levels exist for the two tumor types. Thus, adjustment of tumor ^18^ F-FDG uptake for blood glucose level should be applied only for tumor types that have their ^18^ F-FDG uptake sensitive to blood glucose levels. Moreover, partial volume and glucose-corrected ^18^ F-FDG *K*_*i*_ did not show any significant increase as the tumor of either type grew in size.

## Competing interests

The authors declare that they have no competing interests.

## Authors' contributions

WS participated in conceiving the study, performed the animal experiments and data analysis, and drafted the manuscript. HY participated in performing the animal studies, and KSI participated in the tumor implant and design of experiments. KPW participated in the animal studies and data analysis. MQW performed the statistical analysis. DS participated in performing the animal imaging studies, and WM participated in conceiving the study and in its design. SCH conceived the study, participated in its design, guided the data analysis, and helped draft the manuscript. All authors read and approved the final manuscript.
